# Mutational screening of *SLC39A5*, *LEPREL1* and *LRPAP1* in a cohort of 187 high myopia patients

**DOI:** 10.1038/s41598-017-01285-3

**Published:** 2017-04-25

**Authors:** Chun-Yun Feng, Xiao-Qiong Huang, Xue-Wen Cheng, Rong-Han Wu, Fan Lu, Zi-Bing Jin

**Affiliations:** The Eye Hospital of Wenzhou Medical University, The State Key Laboratory Cultivation Base and Key Laboratory of Vision Science, Ministry of Health, Wenzhou, 325027 China

## Abstract

High myopia (HM) is a leading cause of mid-way blindness with a high heritability in East Asia. Although only a few disease genes have been reported, a small proportion of patients could be identified with genetic predispositions. In order to expand the mutation spectrum of the causative genes in Chinese adult population, we investigated three genes, *SLC39A5*, *LEPREL1* and *LRPAP1*, in a cohort of 187 independent Chinese patients with high myopia. Sanger sequencing was used to find possible pathogenic mutations, which were further screened in normal controls. After a pipeline of database and predictive assessments filtering, we, thereby, identified totally seven heterozygous mutations in the three genes. Among them, three novel missense mutations, c.860C > T, p.Pro287Leu and c.956G > C, p.Arg319Thr in *SLC39A5*, c.1982A > G, p.Lys661Arg in *LEPREL1*, were identified as potentially causative mutations. Additionally, the two heterozygous mutations (c.1582G > A, p.Ala528Thr; c.1982A > G, p.Lys661Arg) in one patient in *LEPREL1* gene were reported in this study. Our findings will not only augment the mutation spectrum of these three genes, but also provide insights of the contribution of these genes to adult high myopia in Chinese. However, further studies are still needed to address the pathogenicity of each of the mutations reported in this study.

## Introduction

High myopia is one of the most severe eye disorders with a strong genetic component^1, 2^. This disease is resulted primarily from excessive axial elongation of the eyeball (longer than 26 mm)^3^, concomitant with obvious refractive error (greater than 6 diopters^4^). It can also predispose the affected individuals to several ocular comorbidities, such as retinal detachment^5, 6^, macular degeneration^7, 8^ and glaucoma^9^. On the other hand, myopia prevalence rates vary and have been increasing worldwide. Multiple studies have shown that its prevalence ranges between 30% to 50% in American, European and Australian populations^10–12^, and is as high as about 71–96% in Asian countries, particularly in China, Singapore and Japan^13–15^.

High myopia has been widely accepted as a complex disorder. Both genetic and environmental factors have been shown to involve in the etiology of myopia^16, 17^. Family and twin studies have indicated that genetic factor, in particular, plays a significant role in the development of high myopia^18, 19^.

Despite intensive study on myopia, its exact molecular mechanism remains unclear, and it is mostly regarded as a polygenic disorder. Genome-wide association studies (GWAS) have mapped several genomic loci associated with myopia to chromosomes 11q24.1^20^, 15q14^21^, 15q25^22^, 5p15^23^, 4q25^24^, 13q12.12^25^in large cohorts. On the other hand, at least 39 susceptibility loci have been identified by linkage analysis for nonsyndromic monogenic myopia^26^. In addition, mutations in six genes associated with high myopia have been detected by next generation sequencing. Of the six, three genes, including *ZNF644* (c.2156A > G, p. Ser672Gly; c.725C > T, p.Thr242Met; c.821A > T, p.Gln274Trp; c.2014A > G, p.Ser672Gly)^27, 28^; *SCO2* (c.157C> T; p. Gln53*)^29^ and *SLC39A5* (c.141C > G; p.Tyr47*, c.T911C; p.Met304Thr)^30^, have been reported for autosomal dominant high myopia, and three other genes have been reported for autosomal recessive high myopia, including *LEPREL1* (c.13C > T, p.Gln5X; c.1523C > T, p.Gly508Val)^31, 32^; *LRPAP1* (c.605delA, p.Asn202Thrfs*8; c.863_864del, p.Ile288Argfs*118)^33^ and *CTSH* (c.485_488del, p. Leu162Profs*66)^33^. Recently, Jiang *et al*.^34^ has identified five novel mutations in several disease-causing genes in 298 unrelated Chinese patients with high myopia, including three heterozygous mutations (p.Lys369Met, p.Ala55OThr and p.Asp851His) in*ZNF644*, a frame shift mutation (p.Gln67Sfs*8) in *LRPAP1* and a heterozygous mutation (p.Gly413Ala) in *SLC39A5*. Up to now, limited mutations in the six causative genes have been confirmed, which contributed to very few high myopia cases being genetically deciphered. In summary of these previous studies, we proposed that those genes mutation exert their characteristics in different regions. *SLC39A5*, *LEPREL1* and *LRPAP1* were more likely to associate with Chinese high myopia patients. Therefore, we screened mutations in the three HM associated genes, *SLC39A5*, *LEPREL1* and *LRPAP1*, and discovered additional mutations in a group of 187 unrelated Chinese patients with high myopia.

## Materials and Methods

### Patient recruitment

A total of 187 patients were enrolled in this study. All patients were clinically diagnosed with high myopia greater than −6.0 D. We selected totally 200 subjects as healthy controls, who met the following criteria: aged more than 60, with no systemic diseases and no high myopia and other known ocular diseases. Written informed consents were obtained from all the participants or their statutory guardians prior to the collection of their genomic DNA. The study was conducted in adherence to the tenets of the Declaration of Helsinki, and was approved by the Ethics Committee of the Eye Hospital of Wenzhou Medical University.

### Mutational screening

Sanger sequencing was used as a direct and rigorous method to identify potential mutations in all three genes, the reported novel variants were further screened in matched controls. In detail, genomic DNA was extracted from leukocytes in the subjects’ peripheral venous blood using the Blood DNA Mini Kit (Simgen, Hangzhou, China) according to the manufacturer’s recommendations, and finally dissolved in TE buffer. PCR primer pairs were designed using the online program ExonPrimer^35^ (http://genome.ucsc.edu/cgi-bin/hgBlat) to amplify all coding regions and intron boundary of *SLC39A5*, *LEPREL1* and *LRPAP1*. The seven primer pairs amplified sequences harboring the mutation were provided in Table 1. All amplified products were separated with polyacrylamide gel electrophoresis. Sequencing was performed with ABI 3730XL automated DNA sequencer (Thermo Fisher Scientific, Carlsbad, CA, USA). The sequences were compared against the known reference sequences obtained from the UCSC genome browser hg19^36^ (http://genome.ucsc.edu/cgi-bin/hgGateway) in order to retrieve and identify SNPs, insertions or deletions. All mutations (Table 2) were screened in 200 healthy controls.

**Table 1:** PCR Primers for Sequences Harboring the Mutations in Present Study.

Primer name	Forward	Reverse
Chr3:189972991	TCAATGCAAGCTAGTGCCTG	TTTGCCTTGTTTCATTTCCC
Chr3:189681799	AGCCAGAGAAGCAGGAGTTG	TTCTTTTCCTCAGACGAAGC
Chr3:190120600	GAGGGAAGGTGGGAGAGG	ACTGAACAGAGATGACGGGG
Chr12:56231524	GATGTTTCGGGGAGAATAGGAG	ATTTGTAACTCCAGGGATCTCG
Chr12:56629399	AGTAGAGCATATGAGCGAAGGC	CAGTTCTTGACTGGGACTCTGG
Chr12:56630190	GTGGAACCAGGTGTTCATCTTC	CAGCTGATAACTAGGAGCCCTG
Chr4:3514801	GTCCTTGCAGTTCACCCG	CGGCCTCATCTTTCCTGC

**Table 2:** Summery of Mutations in *LEPREL1*, *SLC39A5* and *LRPAP1*.

Chr.position	Gene	Exon	Mutation	Status	Patient	Mutation	SIFT	PolyPhen2	PROVEAN	Mutation	Note	ExAC
						Taster				Assessor		
Chr12:56231524	*SLC39A5*	4	c.250C > T (p.Arg84Trp)	Het	HM_h16	DC (0.935)	D (0.004)	Pr.D (0.995)	N (−1.97)	L (1.61)	rs199681035	0.0001771
Chr12:56629399	*SLC39A5*	8	c.860C > T (p.Pro287Leu)	Het	HM_95	DC (0.995)	D (0.047)	B (0.231)	N (−0.57)	N (0.555)	Novel	0.0001895
Chr12:56630190	*SLC39A5*	9	c.956G > C (p.Arg319Thr)	Het	HM_71	P (0.283)	D (0.022)	B (0.174)	N (−0.84)	L (1.09)	Novel	—
Chr3:190120600	*LEPREL1*	1	c.132C > A (p.Phe44Leu)	Het	HM_101	DC (0.996)	T (0.191)	B (0.002)	N (−1.58)	L (1.59)	rs367659257	0.0001129
Chr3:189972991	*LEPREL1*	11	c.1582G > A (p.Ala528Thr)	Het	HM_68	DC (0.999)	D (0.014)	Pr.D (0.977)	D (−3.55)	M (2.83)	rs199877373	0.000132
Chr3:189681799	*LEPREL1*	14	c.1982A > G (p.Lys661Arg)	Het	HM_68	DC (1)	T (0.594)	B (0.000)	N (1.06)	N (−0.22)	Novel	3.30E-05
Chr4:3514801	*LRPAP1*	7	c.962 G > A (p.Arg321His)	Het	HM_h32	P (0.016)	T (0.17)	Pos.D (0.884)	N (−0.60)	M (2.2)	rs140947105	0.00015

Notes: Het, heterozygous; Mutation taster (DC, disease-causing; P, polymorphism); SIFT (D, damaging; T, tolerated); PolyPhen2 (Pr.D, probably damaging; pos.D, possible damaging; B, benign); PROVEAN (D, deleterious; N, neutral); MutationAssessor (M, medium; L, low; N, neutral).

### Bioinformatics analysis

We evaluated all identified mutations using the following software and online database^37^. Polymorphism Phenotyping v2^38^ (PolyPhen-2, http://genetics.bwh.harvard.edu/pph2), Sorting Intolerant from Tolerant^39^ (SIFT, http://sift.jcvi.org) and Mutation Taster^40^ (http://www.mutationtaster.org/) were employed to assess protein structure/function and evolutionary conservation. PROVEAN^41^ (http://provean.jcvi.org/index.php) was used to align and measure the similarity between mutation sequence and protein sequence homologs. Mutations detected in potential splice-sites were analyzed by Human Splice Finder^42^ (HSF, www.umd.be/HSF). SNPs minor allele frequency (MAF) was evaluated by 1000 Human Genome Project^43^ (ftp://1000genomes.ebi.ac.uk/vol1/ftp) and Exome Aggregation Consortium^44^ (ExAC, http://exac.broadinstitute.org/). Mutation assessor^45^(http://mutationassessor.org/) was used to predict the effect of evolutionary conservation.With Clustal Omega^46^ (http://www.ebi.ac.uk/Tools/msa/clustalo/), we also acquired multiple-sequence alignment of *SLC39A5*, *LEPREL1* and *LRPAP1* in different species, including Homo sapiens, Pan Troglodytes, Macacamulatta, Bostaurus, Feliscatus, Mus musculus, Gallus gallss and Danio rerio. SMART^47^ (http://smart.embl-heidelberg.de/) was used to simulate the topological model of the relative genes polypeptide. Furthermore, associated crystal structures of mutant and wild-type proteins were predicted by Phyre2^48^ (http://www.sbg.bio.ic.ac.uk/phyre2/html/page.cgi? id=index) and then visualized by Pymol Molecular Graphics System (Pymol)^49^.

### Mutation criteria

Mutations identified in the three genes from all subjects with high myopia were filtered by the following criteria^34, 50^:

1. Variants in noncoding region that did not affect splicing sites based on prediction of the Berkeley Drosophila Genome Project (http://www.fruitfly.org/) were excluded;
2. Synonymous mutations in genes that did not alter splicing sites were subtracted;
3. Mutations with minor allele frequency (MAF) less than or equal to 0.01 in the Exome Aggregation Consortium (ExAC) were extracted;
4. Nonsynonymous single nucleotide mutations predicted to be benign by three commonly used silico tools (Mutation Taster, SIFT and Polyphen-2) were excluded;
5. Mutations were verified using dbSNP146 and those without *rs* number, were regarded as novel rare mutations.

## Results

**Figure 1:**
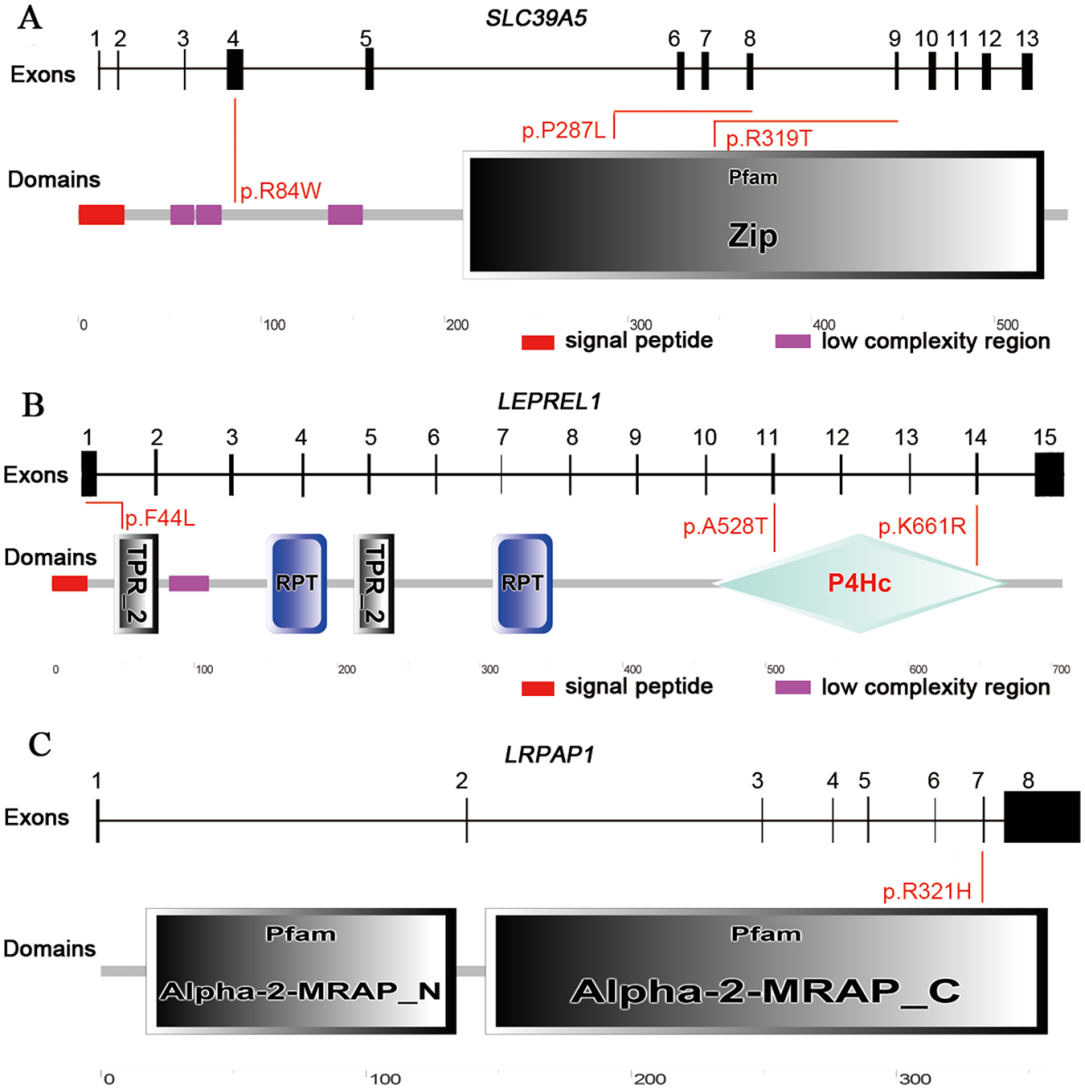
Location of the identifiedmutations in *SLC39A5*, *LEPREL1* and *LRPAP1*. Exons of human *SLC39A5*, *LEPREL1* and *LRPAP1* (upper), and positions of mutated residues corresponding to the topological model of the polypeptides (under). A total of seven missense mutations colored red were identified in this study. All mutations were located in the functional domains, except for the heterozygous mutation c.250C > T (p.Arg84Trp) in *SLC39A5* (A,B,C). Pfam ZIP domain is responsible for metal ion transmembrane transporter activity (**A**). Proteins containing TPRs are involved in many biological processes, such as cell cycle regulation, mitochondrial and peroxisomal protein transport, neurogenesis and protein folding, RPT is an internal repeat, P4Hc domain participatesin inoxidoreductase activity (**B**). Alpha-2-MRAP isa Pfam domain that binds to the alpha-2-macroglobulin receptor (**C**).

We screened for mutations in *SLC39A5*, *LEPREL1* and *LRPAP1* in a cohort of 187 high myopia patients with Sanger sequencing. A total of seven heterozygous mutations from six subjects were confirmed (Fig. 1) by applying the filtering criteria described in the Methods section. All mutations were located in the functional domains, except for the c.250C > T, p.Arg84Trp in *SLC39A5*, according to the prediction of SMART (Fig. 1A–C). None of these mutant alleles were detected in the control population.

### *SLC39A5*Mutations

**Figure 2:**
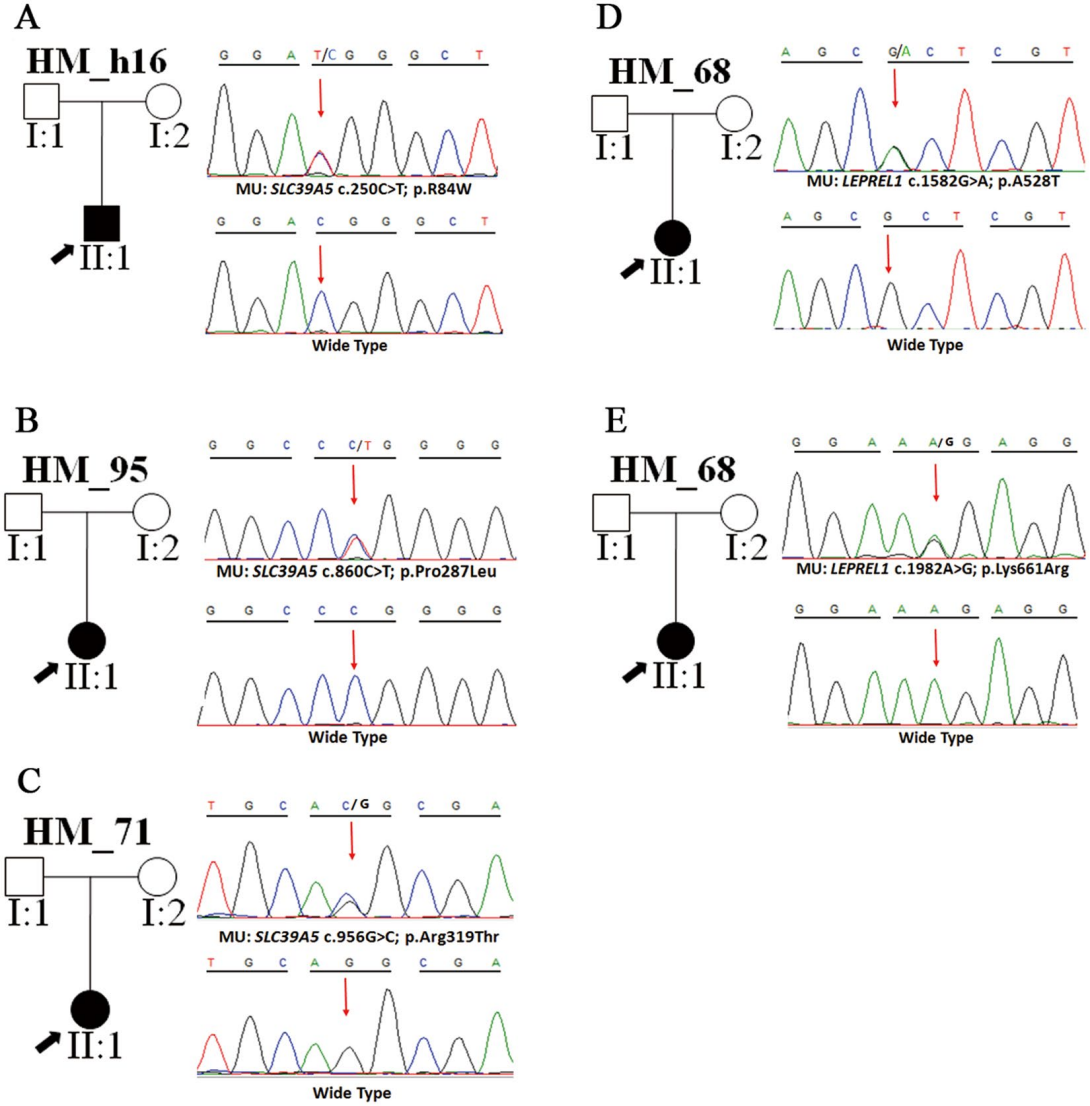
Potentially pathogenic mutations detected in this study. Pedigree plots of mutations. The black arrow represents the patient (left). Sequence profiles of identified mutations and wild type (right) were also shown (**A**–**E**).

**Figure 3:**
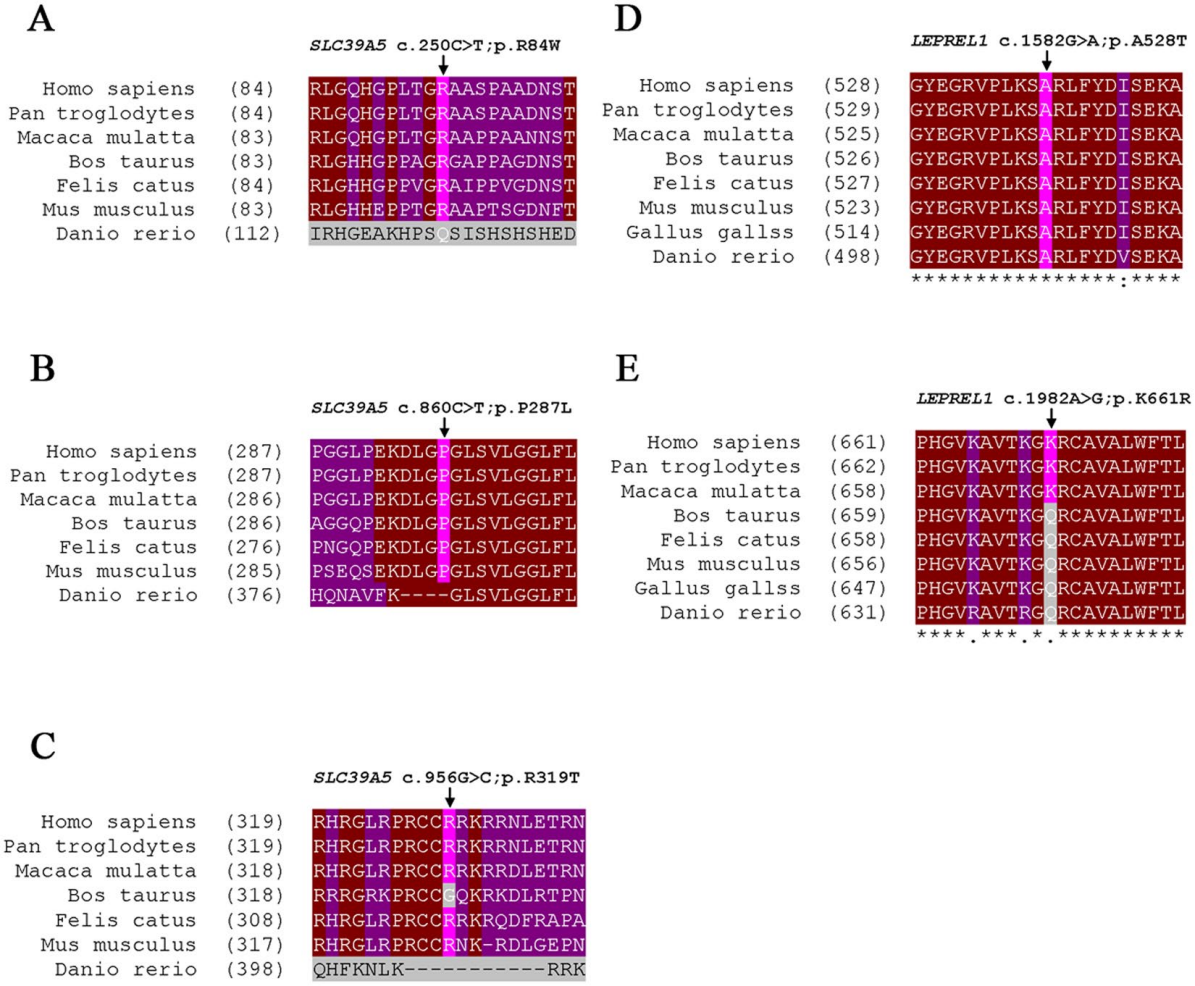
Conservation analysis revealed evolutionary conservation of the mutations. Clustal Omega results showing multiple alignments of the amino acids from different species. The arrow indicates the location of the mutations (**A**–**E**).

**Figure 4:**
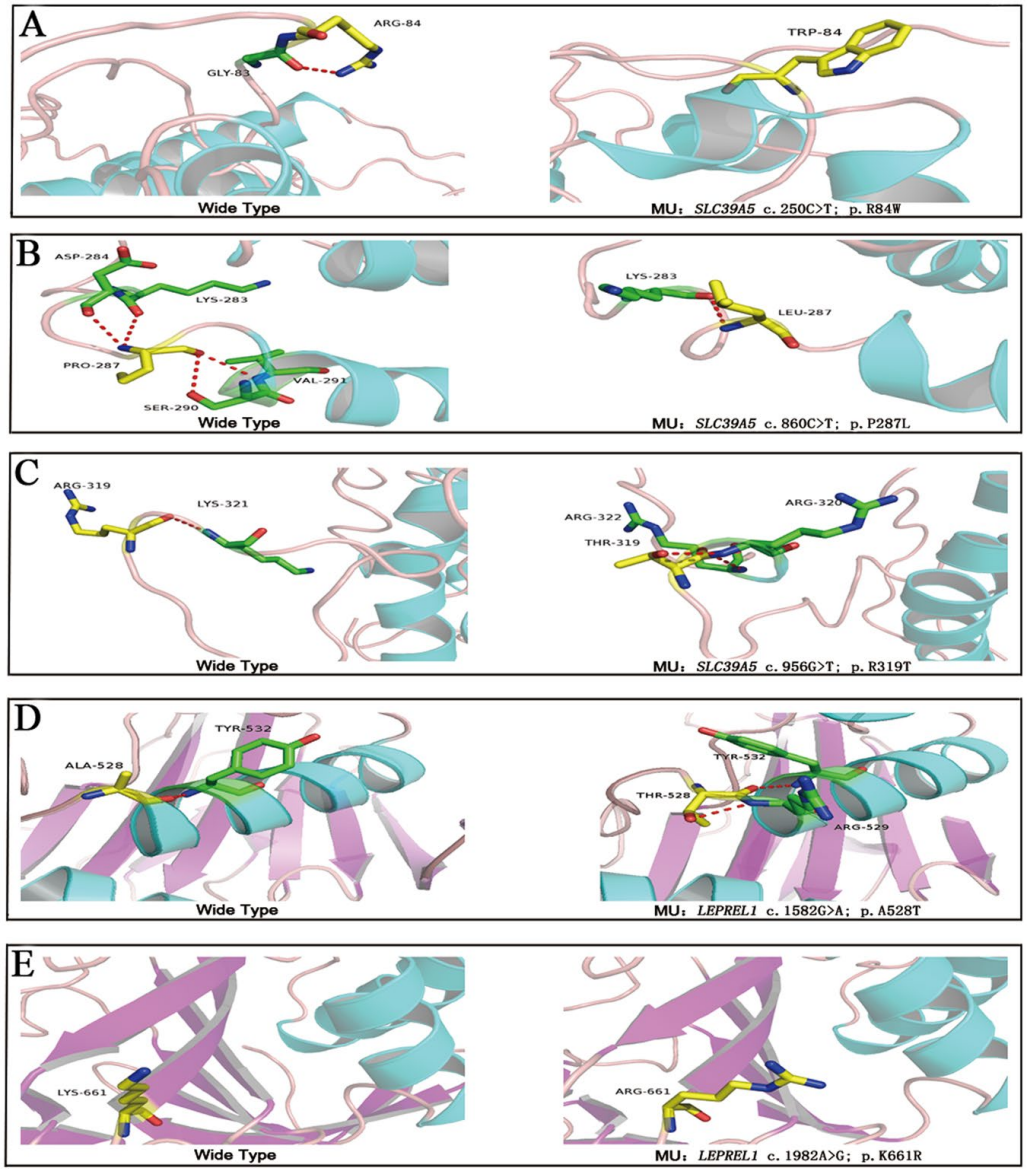
Predicted three-dimensional structure of proteins. Predicted crystal structures of wild type (left) and mutant (right) proteins. Yellow represents residue of wild type (left) and mutant (right), while green indicates residues that interact with wild-type (left) and mutant residue (right) (**A**–**E**).

Three heterozygous mutations were detected in *SLC39A5* (c.860C > T, p.Pro287Leu; c.956G > C, p.Arg319Thr; c.250C > T, p.Arg84Trp) from three sporadic cases (Fig. 2A–C), among which p.Pro287Leu and p.Arg319Thr were novel (Table 2).The substitution p.Pro287Leu was predicted to be pathogenic by both SIFT and Mutation Taster at a low allele frequency. Besides that, mutated amino acid is evolutionarily highly conserved among all the tested species apart from danio after multiple orthologous sequence alignment (Fig. 3B), illustrating that it is important for protein function. Consequently, structural modeling demonstrated the absence of bonds between the mutated residue 287 leucine and residue 284 asparticacid, 290 serine, 291 valine (Fig. 4B). The mutation p.Arg319Thr was predicted to be damaging by SIFT (Table 2). In addition, 3D modeling demonstrated a newly formed bond between residue 319 and residues 320, 322 (Fig. 4C). The mutation p.Arg84Trp caused a substitution of arginine to tryptophan at position 84. This was predicted as damaging by 3 different *in silico* tools (PolyPhen-2, SIFT and Mutation Taster). This mutation was very rare in the ExAC database (Table 2). Furthermore, R84Wwas shown to affect highly evolutionarily conserved amino acid residues (Fig. 3A). In addition, 3D structural modeling revealed the absence of a bond between the mutated tryptophan at residue 84 and glycine at residue 83 (Fig. 4A).

### *LEPREL1* Mutations

In *LEPREL1*, three heterozygous mutations were detected in two isolated individuals. Among them, two heterozygous mutations were found in one patient (Fig. 2D,E). The c.1982A > G, p.Lys661Arg and c.1582G > A, p.Ala528Thr located in exon14 and exon11, respectively. The heterozygous p.Lys661Arg was a novel conservative mutation (Table 2) (Fig. 3E) that was predicted to be disease-causing by Mutation Taster. The p.Ala528Thr has been annotated as an exceedingly rare SNP (rs199877373), and it was predicted as a pathogenic mutation by all 5 pathogenicity prediction tools used in this study (PolyPhen-2, SIFT, Mutation Taster, PROVEAN and Mutation Assessor). Furthermore, the A528T mutation occurred at a remarkably conserved region in various species (Fig. 3D, Table 2), and was projected to produce a substituted bond between the mutated residues 528 and 529, in the 3D protein model (Fig. 4D). The third mutation c.132C > A, p.Phe44Leu was highly conserved among the different species (Figure S1A–C). Nevertheless, it was excluded based on the inheritance pattern of the gene. As expected, the mutation p.Phe44Leu was estimated as benign by both Polyphen-2 and SIFT (Table 2), and no noticeable fundamental changes were observed in protein modeling (Figure S1D, E).

### *LRPAP1* Variant

A heterozygous variant c.962G > A, p.Arg321His was detected in a HM patient (Figure S2A,B). It was predicted to be a benign polymorphism by both SIFT and Mutation Taster (Table 2). Likewise, R321H was excluded according to the inheritance pattern of the gene and its resulting change in residues showed no effect on crystal structure modeling (Figure S2 C–E).

## Discussion

To date, tremendous efforts have been made to better understand the genetics of high myopia. However, only a few studies that explored mutations in the six causative genes (*SLC39A5* ^30, 34^, *LEPREL1* ^31, 32^, *LRPAP1* ^33, 34^, *CTSH* ^33^, *SCO2* ^29, 34^ and *ZNF644* ^27, 34^) have been reported. In this study, we attempted to replicate previous results and broaden the mutation spectrum of HM associated genes in a Chinese high myopia cohort. Based on previous reports, we proposed that *SLC39A5*, *LEPREL1* and *LRPAP1*were more likely to associate with Chinese high myopia patients. We regarded them as preferential genes, and set out to screen mutations in these three genes using Sanger sequence. We identified a total of seven mutations that were predicted to influence the functional residues. These included three heterozygous mutations in *SLC39A5* (p.Pro287Leu; p.Arg319Thr and p.Arg84Trp) from three sporadic cases, three heterozygous mutations (p.Lys661Arg; p.Ala528Thr and p.Phe44Leu) in *LEPREL1* in two isolated individuals, a heterozygous mutation p.Arg321His in *LRPAP1*. Among these seven mutations, three mutations, p.Pro287Leu and p.Arg319Thr in *SLC39A5* and p.Lys661Arg in *LEPREL1*, have not been previously reported. Our results were consistent with those reported in previous study in that missense mutations accounted for the largest proportion of the four mutation types in reported monogenic high myopia patients^50^.

*SLC39A5* encodes the solute carrier family 39, member 5, which is a key member of the ZIP transporters for metal ions, especially in mammalian zinc omeostasis^51^. *SLC39A5* has also been shown to express in all developmental stages of mouse ocular tissues, and is especially abundant in the sclera and in several layers of the retina. In addition, *SLC39A5* might be involved in high myopia by regulating BMP/TGF-β, and the disruption of this pathway may be the underlying mechanisms of high myopia^30^. Guo *et al*. first identified that mutations in *SLC39A5* were associated with autosomal dominant high myopia in two Chinese family, which included a nonsense mutation (c.141C > G, p.Tyr47*) and a missense mutation (c.911T > C, p.Met304Thr)^30^. Jiang *et al*. subsequently reported another heterozygous missense mutation c.1238G > C, p.Gly413Ala in a sporadic case^34^. In this study, three missense mutations p.Arg84Trp; p.Pro287Leu and p.Arg319Thr were detected in three isolated patients, and two of them have never been reported. Through secondary structure modeling, we found a novel mutation p.Pro287Leu, which was locatzed in the Pfam ZIP domain of the SLC39A5 (Fig. 1A). We modeled the 3D structure of SLC39A5 protein by applying Phyre2 program^52^. The structural modeling showed an absence of bond between the mutated residue 287 leucine and the residues 284 asparticacid, 290 serine and 291 valine (Fig. 4B). Since residue 290 was predicted to be a phosphorylation site, the p.Pro287Leu mutation may affect phosphorylation events. Further functional studies are needed to elucidate the molecular mechanism of high myopia as related to *SLC39A5*, however, our results may provided additional genetic evidence for potential contribution of *SLC39A5*in high myopia.

*LEPREL1*encodes a member of the prolyl 3-hydroxylase subfamily of 2-oxo-glutarate-dependent dioxygenase (P3H2). Expression of *LEPREL1* has been detected in various collagen fibril-containing tissues, including the eye^53^. As reported, *P3h2* ^*n/n*^mouse suffered from a significant defect in collagen prolyl 3-hydroxylation compared with their wild type littermates, which led to result in structural abnormalities in multiple eye tissues. These findings suggested that altered collagen hydroxylation caused by loss of LEPREL1 can potentially contribute to the myopia^54^.Mordechai *et al*.^31^ previously identified a homozygous mutation c.1523G > T, p.Gly508Val in *LEPREL1*from affected individuals in a Bedouin kindred. Co-segregation of one homozygous nonsense mutation in *LEPREL1* (c.13C > T, p.Gln5*) with autosomal-recessive high myopia was reported in Chinese family by Guo *et al*.^32^. In this study, we identified two heterozygous mutations p.Ala528Thr and p.Lys661Arg in one high myopia patient. Of note, we also found a heterozygous mutation p.Phe44Leu in *LEPREL1*, which was excluded in this study due to the inheritance pattern of the gene.

*LRPAP1*encodes a Low Density Lipoprotein (LDL) Receptor-Related Protein Associated Protein 1, a 357 amino acid protein that binds and protects the LDL receptor-related protein (LRP1), which is known to influence transforming growth factor-βactivity^33, 55, 56^. Furthermore, TGF-β signal pathway has been highlighted as the responsible factor for regulation of scleral metabolism in myopia^57^. Therefore, *LRPAP1* may affect the formation of myopia by regulating TGF-β signaling. So far, three homozygous frame shift mutations (Asn202Thrfs*8; Ile288Argfs*118; Gln67Serfs*8) in*LRPAP1*have been reported to associate with autosomal recessive high myopia in Arabic and Chinese families^33, 34^. Here, we detected a heterozygous mutation p.Arg321His in *LRPAP1*. However, p.Arg321His already existed in the database (rs140947105) and was excluded based on the inheritance pattern of this gene. Nevertheless, co-segregation analysis could not be performed due to the unavailability of familiar samples. In addition, functional consequences of these mutations need to be investigated in future studies.

In conclusion, we carried out mutational screening in three causative genes in a large cohort of high myopia patients. We identified seven mutations, including three novel mutations. Our findings widen the mutation spectrum of known HM-genes and provided additional genetic evidence that*SLC39A5* and *LEPREL1*may be associated with high myopia in Chinese population. Nevertheless, given the paucity of our data on the pathogenicity of these genes, further studies are needed to better understand the potential roles of these genes in the development of high myopia.

## Electronic supplementary material

Supplementary information
