# Supplementary material for: Mutational screening of SLC39A5, LEPREL1 and LRPAP1 in a cohort of 187 high myopia patients
Source: Sci Rep. 2017 Apr 25;7:1120. doi: 10.1038/s41598-017-01285-3 (PMC5430800; doi:10.1038/s41598-017-01285-3)
Supplement: Supplementary file 1 — Supplementary information [file 41598_2017_1285_MOESM1_ESM.docx]

**Mutational screening of *SLC39A5*, *LEPREL1* and *LRPAP1* in a cohort of 187 high myopia patients**

Chun-Yun Feng^1†^, Xiao-Qiong Huang^1†^, Xue-Wen Cheng^1^, Rong-Han Wu^1^, Fan Lu^1^, Zi-Bing Jin^1^

1. The Eye Hospital of Wenzhou Medical University, The State Key Laboratory Cultivation Base and Key Laboratory of Vision Science, Ministry of Health, Wenzhou 325027, China;

† These authors contributed equally to this work.

***Correspondence**: Dr. Zi-Bing Jin, The Eye Hospital of Wenzhou Medical University, The State Key Laboratory Cultivation Base and Key Laboratory of Vision Science, Ministry of Health, Wenzhou 325027, China. E-mail: jinzb@mail.eye.ac.cn. Tel/fax: +86-577-88067926.

**Competing interests**

The authors have declared that no competing interests exist.

**The Supplementary information included Supplementary Figure 1, 2.**

**
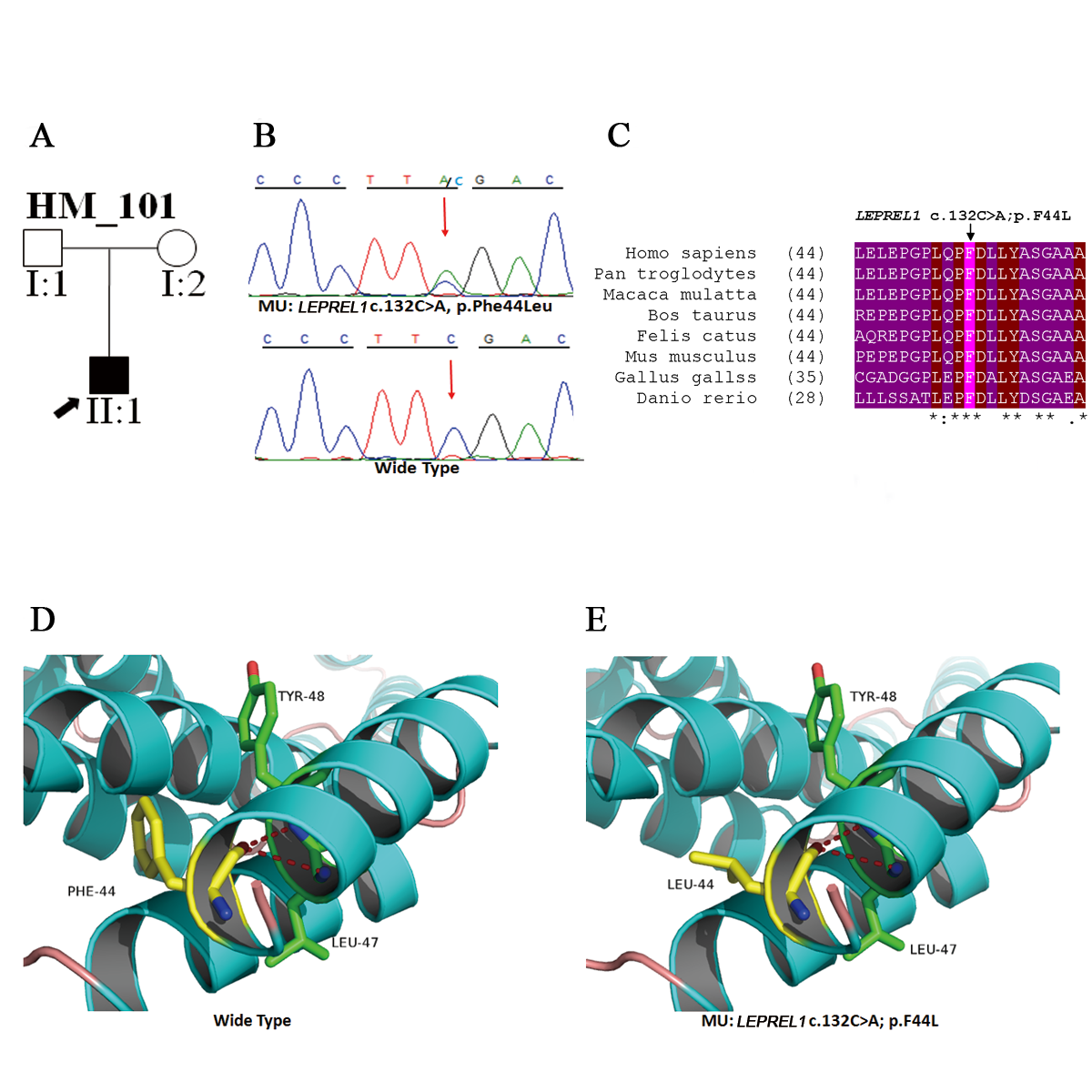
**

**Figure s1. Analysis of the mutation c.132C>A (p.Phe44Leu) in *LEPREL1.*** Pedigree and a heterozygous mutation c.132C>A (p.Phe44Leu) discovered by Sanger sequencing, WT sequence was also shown (A, B). Multiple sequence alignment indicates an extremely high level of sequence conservation(C).Structural modeling of wild type and mutant LEPREL1 protein (D, E).

**
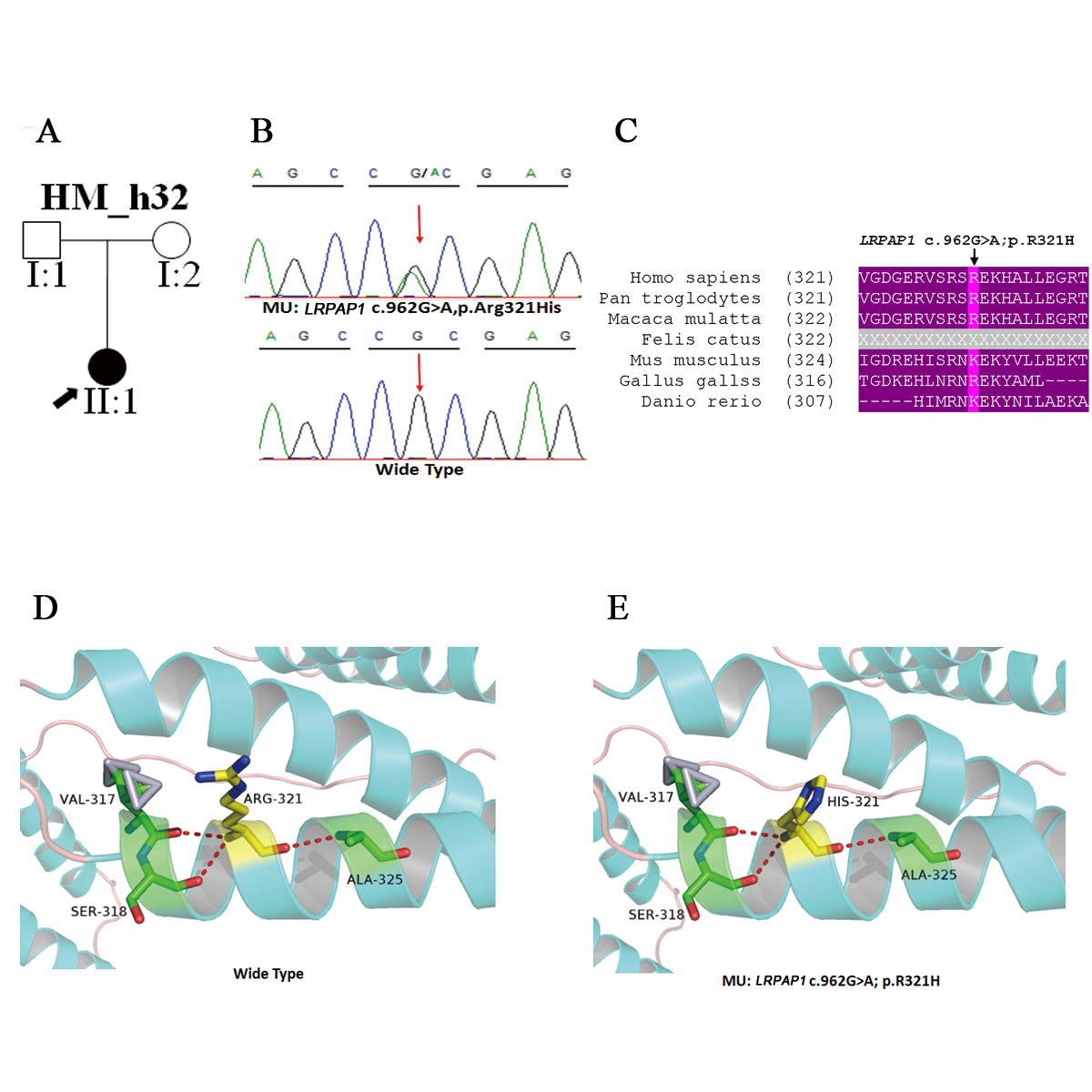
**

**Figure s2. Analysis of the mutation c.962G>A (p.Ala321His) in *LRPAP1.*** Pedigree plot of mutation and sequencing results of mutant and wild type (A, B). p.Ala321His was not conserved throughout evolution and residue changes had no effect on crystal structure modeling (C-E).
